# Compositional characteristics of dairy products and their potential nondairy applications after shelf-life

**DOI:** 10.1016/j.crfs.2021.12.017

**Published:** 2022-01-06

**Authors:** Nermeen N. Nasralla, Nanis H. Gomah, Morsy M. Aly, Jelan A. Abdel-Aleem, Ahmed R.A. Hammam, Dina M. Osman, Yaser M.A. El-Derwy

**Affiliations:** a Dairy Science Department, Faculty of Agriculture, New Valley University, El-Kharga, 72511, Egypt; b Dairy Science Department, Faculty of Agriculture, Assiut University, Assiut, 71526, Egypt; c Faculty of Science, University of Hafr Al Batin, Hafar Al-Batin, 31991, Saudi Arabia; d Department of Chemistry, Faculty of Science, Assiut University, Assiut, 71526, Egypt; e Department of Industrial Pharmacy, Faculty of Pharmacy, Assiut University, Assiut, 71526, Egypt; f Dairy and Food Science Department, South Dakota State University, Brookings, SD, 57007, USA

**Keywords:** Expired dairy products, Shelf-life, Gas chromatography, Electrophoresis analyses, Cosmetic creams, Soap

## Abstract

Many dairy products are discarded and useless after end of shelf-life, which causes economic and environmental challenges. The objective of this study was to study the compositional characteristics of some dairy products before and after shelf-life, and develop a process to utilize those dairy products after end of shelf-life in non dairy applications (cosmetic cream and soap). Several dairy products, such as sterilized milk, yogurt, soft cheese, hard cheese, cream, and butter were collected from markets in Egypt before shelf-life and after three months of shelf-life. Electrophoresis analysis was conducted to estimate the changes in the protein fractions of protein products (sterilized milk, yogurt, and cheese) before and after expiration. Also, gas chromatography (GS) was performed to compare the fatty acids of fat products (cream and butter) before and after end of shelf-life. Sterilized milk, yogurt, soft, and hard cheese were turned into powder (Expired dairy products powder; EDPP) to be used as a raw material in manufacturing of cosmetic creams. The fat was separated from cream, butter, and hard cheese (Expired dairy products fat; EDPF) to be utilized in making soap. The formulated cosmetic creams were examined in vitro. Functional properties of cream were determined, such as appearance, spreadability, irritancy, and pH. Additionally, the soap quality was tested after manufacture. We found that dairy products, such as milk, yogurt, and cheese after shelf-life can be utilized as raw materials for the production of cosmetic creams, as well as production of soap from butter and cream. The produced products were similar to those in commercial markets. This study is an endeavor to conquer the dairy industry challenges, which are considered a huge loss from the economic and environmental aspects.

## Highlights

• Several dairy products were collected from markets before and after shelf-life.
• The protein fractions were estimated in those dairy products before and after expiration.
• Sterilized milk, yogurt, soft, and hard cheese after end of shelf-life were turned into powder.
• This powder can be used as a raw material in manufacturing of cosmetic creams.
• The fat of expired dairy products was used in making soap.

## Graphical abstract

**Figure:**
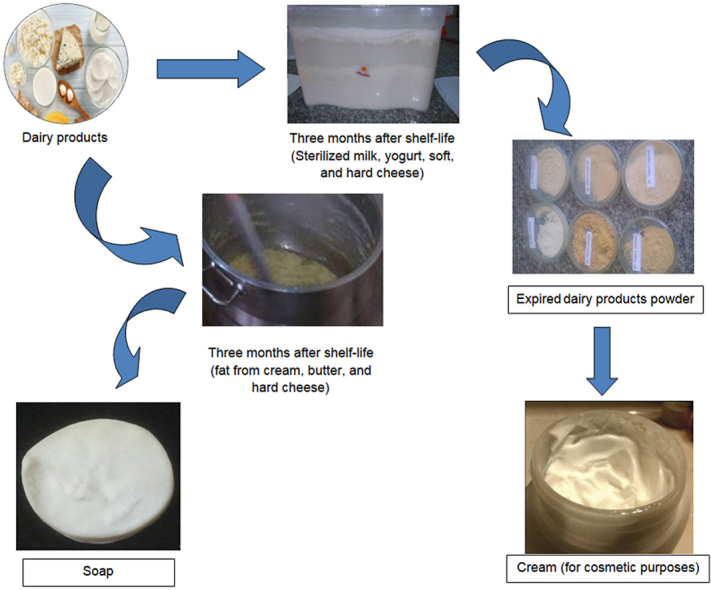

## 1 Introduction

The shelf-life of a dairy product is limited by the growth of spoilage bacteria (Muir, 1996a), which produces enzymes that can degrade milk components and cause unacceptable quality (Amelia and Barbano, 2013; (Hammam et al., 2021); Hammam, 2019). Milk fatty acid composition is a key factor determining its storage condition (Sommella et al., 2018), where lipids in dairy products change during storage. This can reduce the shelf-life and quality of milk and alter the overall flavor (Ma et al., 2000). For these reasons, milk is increasingly subjected to quality control and safety assessment (Grumetto et al., 2013) through the evaluation of the chemical composition, as well as levels of different microorganisms, which are responsible for the lipolysis, lipid oxidation, and milk spoilage.

The shelf-life is an indefinite term and difficult to measure with accuracy. Inaccurate detection methods for milk spoilage force milk producers to use excessively conservative expiration dates to avoid the legal and economic consequences for consumers from drinking spoiled milk (Lu et al., 2013). The stability of a product is defined as the time during which the product remains safe and does not exhibit any sensory or organoleptic defects (Muir, 1996a). These dates estimate of the shelf-life of dairy product, which are imprecise due to variable processing, shipping, and storage conditions (Lu et al., 2013). However, in most cases, a product's shelf-life refer to the quality of the product that may deteriorate after a certain time.

In Egypt, any product that exceeded shelf-life (as shown on the label) is considered no longer suitable for human consumption. Consumers determine dairy products' shelf-life by checking the "sell by" and or "best if used by" dates on the labels. They do not prefer to buy products that about to be expired (Kim et al., 1997). In other foreign countries, it is the responsibility of the manufacturer, not the government to set product quality standards.

Codex refers to shelf-life as “date of minimum stability” which means the same as (“best before”). The date signifies the end of the period under any stated storage conditions, in which the product remains fully marketable and maintains its characteristics. However, beyond the date, the food may still be perfectly satisfactory (Codex General Standard, 1985). As a result, in Codex, the date of minimum stability represents end of the marketing life and is appropriately labeled “best before”. The disposal of expired dairy products is one of the crucial challenges that have significant effects on human health and the environment (WHO, 2015). Additionally, expired dairy products are a major problem for the dairy industry from the economic aspects. There are different disposal methods for expired dairy products, including discarding in permitted wastewater treatment stations and discharge to a permitted sanitary sewer, which is costing more, as well as, causes environmental pollution (Sommella et al., 2018). This led to consider new methods to recycle these products after shelf-life, which can be optimal for the dairy and food industries from the environment and economic aspects.

The total world milk production was about 915 million tons in 2018. Developed countries give one-third of world milk production (FAO, 2018), where milk is considered an essential food for poor families. Egypt shared about 16.4% of the Middle East dairy market in 2010. Egypt annually produces 32.3, 4.1, and 2.8 million tons of fresh milk, cheese, and yogurt, respectively (FAO, 2018). This indicates that the quantity of expired products is not small. Expired dairy products contain important components with high economic value, such as protein, fat, and lactose. Milk has essential components approximately 12.9, 4.3, 3.4, 0.7, and 4.4% of total solids, fat, protein, ash, and lactose, respectively (Hammam et al., 2020), which can be used as raw materials in many applications.

Fat can be extracted from expired dairy products (EDPF) especially fatty products, such as cream and butter (Sommella et al., 2018) to manufacture soap (Muir, 1996b; Robertson, 2006; Warra, 2013; Ashraf et al., 2016). Soaps are sodium or potassium salts and long-chain fatty acids found in plants and animals. Potassium soaps (soft soaps) tend to be liquids and are used in shaving creams while those containing sodium are usually solid (hard soaps). Soap manufacturing takes place in four steps which are (i) saponification, (ii) removal of glycerin, (iii) soap purification, and (iv) addition of perfumes and color (Ohikhena, 2007). Additionally, protein in sterilized milk, yogurt, soft and hard cheese can be used to produce expired dairy products powder (EDPP) to prepare skin creams. Skin creams are generally oil in water (O/W) or water in oil (W/O) emulsions. No study has reported applications of dairy products after shelf-life. Therefore, this study aims to examine the compositional properties of different dairy products before and after shelf-life, and develop a process to utilize those dairy products after end of shelf-life in non dairy applications (cosmetic cream and soap).

## 2 Material and methods

### 2.1 Experimental design

Sterilized milk, yogurt, soft cheese, hard cheese, cream, and butter samples were obtained from the main stores of Dairy Factories in Egypt. Those products were analyzed before shelf-life. New batches of those products were stored at 4°C until the end of shelf-life, then they were analyzed again after end of shelf-life.

### 2.2 Chemical and microbiological analyses

The total protein using Kjeldahl method (AOAC, 2000; method 991.20; 33.2.11) and multiply the nitrogen content by 6.38, titratable acidity by calculating the total lactic acid (Sadler and Murphy, 2010), and fat using Gerber method (Kleyn et al., 2001) were determined in all products. Lactose content was measured according to the International Standard method in all dairy products samples (AOAC, 2000; method 896.01). The iodine value (AOAC, 2000; method 993.20), saponification number (AOAC, 2000; method 920.160), and soluble volatile fatty acids (AOAC, 2000; method 960.30) were also determined in extracted fat from fat products and hard cheese. The total bacterial count (TBC) was determined as described in other studies (Hammam et al., 2018; Ahmed et al., 2021; Hamdy et al., 2021; Kamel et al., 2021b; a; Mansour et al., 2021).

### 2.3 Electrophoresis analysis

Electrophoretic separation of proteins was performed at a pH of 8.6 using disc electrophoresis (Davis, 2006). Milk, yogurt, and cheese samples were emulsified in 2% sodium citrate solution containing 7.0 M urea (El-Gomhouria co., Cairo, Egypt). The solution was centrifuged at 3000 rpm (BHG 600 centrifuge) for 15 min to remove the fat layer, and the transparent portion was used for electrophoresis analysis. Sodium dodecyl sulfate Polyacrylamide gel was prepared (Schägger and von Jagow, 1987).

### 2.4 Determination of fatty acids

The lipid extraction was done from cream, butter, and hard cheese products using chloroform methanol (United co., Chem & Med Preparations Egypt) at 2:1 v/v (AOAC, 2000; Moneeb et al., 2021). The non-lipids components were removed by washing lipid extract three times with methanol of 1:1 v/v (El-Nasr Pharmaceutical Chemicals co., Egypt). The lipids in chloroform were dried over anhydrous sodium sulfate (El-Gomhouria co., Cairo, Egypt) and then the solvent was removed by heating at 60°C under a vacuum. The crude lipids were kept in brown bottles at 4°C until further analysis. The abovementioned bottles were transferred using an icebox to the Agricultural Research Center, Giza. Gas liquid chromatography (GC) analysis was carried out in the central laboratory of the food technology research institute.

The methyl esters of fatty acids were prepared from aliquots of total lipids using 5 ml of 3% sulfuric acid (El-Nasr Pharmaceutical Chemicals co., Egypt) in absolute methanol and 2 ml benzene (Rossell et al., 1983). The contents were heated for methanolysis at 90°C for 90 min followed by cooling. Subsequently, phase separation was performed by the addition of 2 ml distilled water, and methyl ester was extracted with 2 ml aliquots of 5 ml hexane each. The organic phase was removed, filtered through anhydrous sodium sulfate (El-Gomhouria co., Egypt), and then concentrated by using a rotary evaporator. The methyl esters of fatty acids obtained from lipids of samples and standard materials were analyzed using a PYE Unicam Pro-GC gas liquid chromatography equipped with dual flame ionization and were carried out on SP-2310 column (10% PEGA; polyethylene glycol adipate) on chromosorb W-AW, and packed with 55% cyanopropyl phenyl silicone dimensions. Column temperature: first the temperature was set to increase from 70 to 190°C at a rate of 8°C/min, then isothermal for 10 min at 190°C. The injector and detector temperatures were 250°C and 300°C, respectively. Carrier gas: nitrogen was set at a rate of 30 ml min^-1^, hydrogen flow rate was set at 33 ml min^-1^, and airflow rate was set at a rate of 330 ml min^-1^. The chart speed was 0.4 cm min^-1^. Peak identifications were established by comparing the retention times obtained with standard methyl esters. The areas under the chromatographic peak were measured with electronic integrator.

### 2.5 Expired dairy products powder (EDPP) preparation

Sterilized milk was centrifuged using centrifugal separators (Alfa-Laval 104, made in Sweden, 16000 Rpm), and then skim milk was collected, placed into Petri dishes (7.0 cm diameter) and dried completely in an air forced oven (GOMAC, El-Gomhouria co., Cairo, Egypt) for 36 h at 60°C. Then, dried milk was grinded by a Blender mill and stored in the refrigerator (at 4–7°C) for further analyses.

**Fig. 1:**
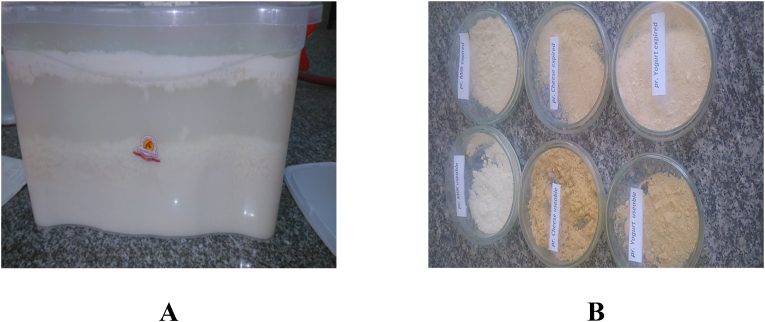
Expired dairy products powder (EDPP) preparation: Products before (A) and after (B) drying.

Yogurt was prepared by using gravity separation methods and diluted with 80°C distilled water in the ratio of 1:3, respectively. The diluted yogurt was then mixed well in a blender and kept at room temperature for about 8 h without stirring. As a result, three different layers (fat, water, and protein) were obtained (Fig. 1A) (Ma and Barbano, 2000). Fat was then separated and collected. Thereafter, the protein was filtered using clean fine clothes to get rid of excess liquid and then dried in the air forced oven at 60°C for about 2 d. The same procedures were applied to the soft and hard cheeses except for the dilution rate (cheese: hot distilled water at a ratio of 1:7). The high levels of fat were separated from hard cheese and collected to be used in soap production. The EDPP was collected and grinded to be used as efficient raw materials for production of therapeutic and cosmetic creams due to its high protein levels (Fig. 1B).

#### 2.5. 1 Chemical analysis of EDPP

Moisture using a forced draft oven (AOAC, 2000; method 990.20; 33.2.44), total protein, and fat were also determined in EDPP produced from milk, yogurt, and cheese.

### 2.6 Expired dairy products fat (EDPF) preparation

**Fig. 2:**
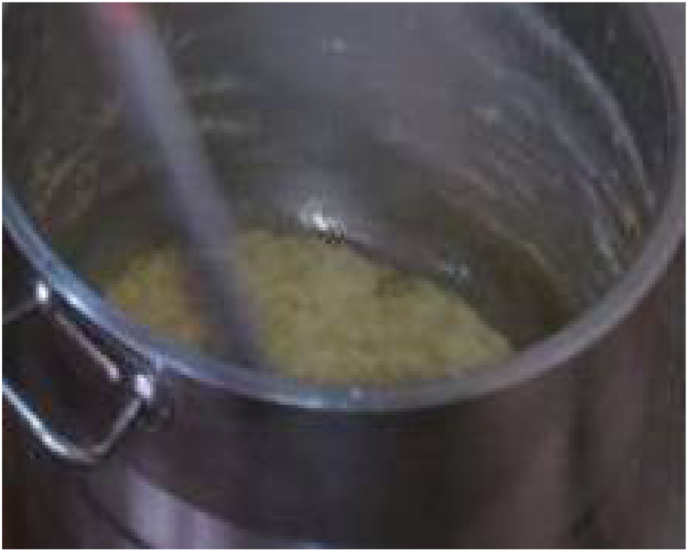
Expired dairy products fat (EDPF) preparation.

Cream and butter were boiled in the pan and stirred continuously until the milk proteins started to coagulate and their color changes from white to golden brown. The endpoint of the boiling stage is similar to the color of ghee (Fig. 2). Then, the EDPF was left to cool and allow particles to settle at the bottom of the pan. After that, the EDPF was filtered carefully using cheesecloth until it became transparent (Sserunjogi et al., 1998). The same procedures were applied to extract fat from hard cheese.

### 2.7 Formulation and evaluation of cold cream

#### 2.7. 1 Formulation of beeswax-borax cold cream

The W/O emulsion based cold cream was prepared, of borax (ISO-CHEM, Egypt) in 2.5 ml water by heating (aqueous phase). Then 1.25 g of beeswax (ISO-CHEM, Egypt), 1.25 g soft paraffin (ISO-CHEM, Egypt), and 5 ml liquid paraffin (ISO-CHEM, Egypt) were added to a porcelain dish (oily phase). The W/O emulsion was prepared by the addition of aqueous phase to the oily phase drop by drop on a constant stirring over a thermostatically controlled boiling water bath with a shaker (Dihan Scientific, WSB 45, Korea). Finally, the dish was removed from the water bath and the dairy product powder was added. Methylparaben (Judex Laboratories Reagent, UK) and rose water (ISO-CHEM, Egypt) were then added and stirred well until semi homogenous cream was obtained and the cream cooled to room temperature (Khan et al., 2010).

#### 2.7. 2 Evaluation of the formulated cream

The cream was evaluated for appearance, spreadability, irritancy test, and pH. The appearance of the cream was judged by its color, texture, roughness, and odor (Khan et al., 2010). The spreadability test was done on the prepared creams by placing samples of 0.25 g from each formula in between two glass slides and left for 5 min to ensure no more spreading. The upper slide was fitted with a string that was tied with a fixed weight. The string was passed over a pulley, and the weight was hung from the string. Under the weight, the upper glass slide took time to slip off so the time was recorded. The short time is taken for the separation of two slides means better spreadability, which was calculated using the following formula (1):

(1) $$S=\frac{MxL}{T}$$

where M = weight tied to the upper slide; L = length of glass slides; T = time taken to separate the slides.

The irritancy test was also measured by marking a 1 cm^2^ area on the left hand surface. A half gram was applied to the specified area. Irritancy, erythema, and edema were monitored for 24 h (Khan et al., 2010). The pH was measured by weighing 1.0 ± 0.01 g of the cream and stirred in distilled water until a uniform dispersion was formed. It was left at room temperature for 2 h. The volume was then adjusted to 100 ml and then the pH was measured (Khan et al., 2010).

### 2.8 Manufacture and characteristics of soap from EDPF

Ten gram of EDPF was heated on a boiling water bath (100°C) for 10 min, followed by adding 4.0 g sodium hydroxide (El-Nasr Pharmaceutical Chemicals Co., Egypt) in 10 ml water with continuous stirring. Saponification takes place gradually in 15 min (200 mg of NaOH used for each 1 kg of EDPF). The thick solution was pulled into a model holder and left overnight to ensure complete saponification. The pH of resulted soap was 11 whereas the typical pH of soap is 9.0. The soap was subjected to washing by dissolving in a minimum amount of water followed by separation through salting using sodium chloride salt three times to decrease the pH.

### 2.9 Statistical analyses

Compositional data were analyzed using R software (R × 64-3.3.3, R Foundation for Statistical Computing) to determine the differences among means of products before and after shelf-life. An ANOVA was performed using the GLM procedure available in R software. When a significant difference (P < 0.05) was detected, differences among means were compared using the LSD test.

## 3 Results and discussion

### 3.1 Composition

The chemical composition and TBC of dairy products before and after shelf-life are shown in Table 1. The acidity of each sample was also measured as an indicator for the quality or deterioration of the product properties. Results indicated that there were no noticeable changes (P > 0.05) in fat, protein, acidity, and lactose contents as well as the TBC of the sterilized milk before and after the expiration date. Also, the data revealed that the values of fat and protein contents in yogurt and both cheese samples did not change (P > 0.05) in good samples compared to samples after end of shelf-life; However, the acidity of those products was high (P < 0.05) after expiration. Lactose did not change much in yogurt and hard cheese but it decreased (P < 0.05) in soft cheeses. The TBC was noticed in both types of cheese but not yogurt. Differences (P < 0.05) were found in the fat of cream and butter but no changes were found in the protein, acidity, lactose, and TBC in these products. acidity values which were slightly increased in some samples refer to the grow of microorganisms that ferment lactose to produce lactic acid, which slightly decreased the lactose content in the expired samples. The TBC was not detected in sterilized milk, yogurt, and cream before and after shelf-life. However, the TBC was shown in soft (3.6 log cfu g^-1^) and hard cheese (3.5 log cfu g^-1^), as well as, in butter (4.5 log cfu g^-1^). Those values have decreased to 3.2, 3.1, and 4.2 log cfu g^-1^ in soft cheese, hard cheese, and butter, respectively.

**Table 1:** Mean (n = 3) chemical composition and total bacterial count (TBC) of dairy products before and after shelf-life.

Product	Components (%)								TBC (log cfu/g)	
	Fat		Protein		Acidity		Lactose			
	Before	After	Before	After	Before	After	Before	After	Before	After
Sterilized Milk	3.00	2.90	3.10	3.00	0.20	0.22	3.70	3.40	0.00	0.00
Yogurt	2.90	3.20	3.70	4.00	0.75^b^	1.38^a^	3.00	2.70	0.00	0.00
Soft cheese	24.4	22.3	17.2	17.0	0.58^b^	0.90^a^	3.90^a^	3.20^b^	3.60	3.20
Hard cheese	28.2	28.5	27.9^b^	28.6^a^	0.80^b^	0.99^a^	1.70	1.70	3.50	3.10
Cream	34.2^a^	33.0^b^	2.90	2.80	0.12	0.16	2.60	2.30	0.00	0.00
Butter	82.5^b^	85.5^a^	0.50	0.45	0.10	0.10	0.20	0.20	4.50	4.20

^a–b^Means in the same row of each category not sharing a common superscript are different (P < 0.05).

The composition of EDPP produced from expired products (milk, yogurt, and a mix of soft and hard cheese) is presented in Table 2. The moisture content was approximately 5.7, 3.6, and 3.8% in milk, yogurt, and cheese mixes, respectively. The protein content in milk, yogurt, and cheese mixes was 12.9, 16.4, and 15.5%, respectively. The fat was higher in yogurt (42.4%) and cheese mixes (38.3%) as compared to milk (12.2%).

**Table 2:** Mean (n = 3) chemical composition of expired dairy products powder (EDPP) made from milk, yogurt, and cheeses.

Composition (%)	Milk	Yogurt	A mix of soft and hard cheeses
Moisture	5.74	3.64	3.79
Protein	12.86	16.40	15.50
Fat	12.17	42.42	38.33

### 3.2 Milk fat constants in fatty products

**Fig. 3:**
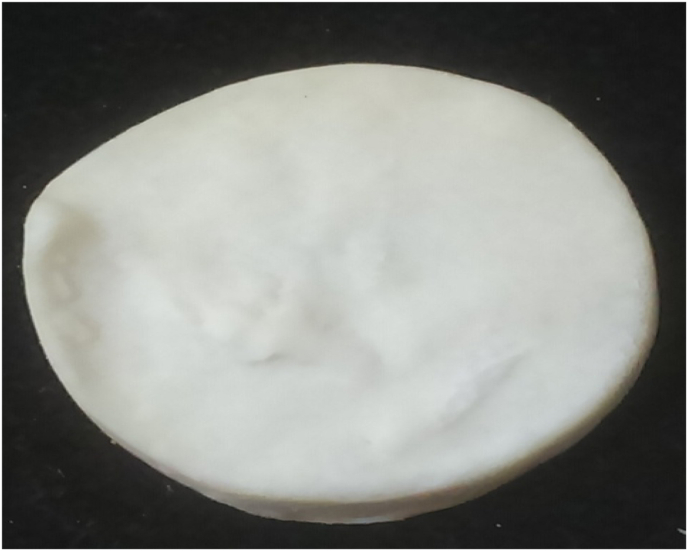
Soap produced from expired dairy products fat (EDPF).

The iodine value, saponification number, and free fatty acids in cream, butter, and hard cheese before and after expiration are presented in Table 3. No significant differences were found in iodine values, saponification number, and free fatty acids among different samples before and after expiration. This indicates the efficiency of the soap manufactured (Fig. 3)

**Table 3:** Mean (n = 4) of iodine, saponification, and free fatty acids values in fat products before and after shelf-life.

Milk fat constants	Cream Fat		Butter Fat		Cheese Fat	
	Before	After	Before	After	Before	After
Iodine values (g 100^-1^ g)	24.0	24.0	30.0	29.0	26.0	26.0
Saponification number (mg KOH g^-1^)	227.0	229.0	237.0	238.0	225.0	233.0
Free fatty acids	28.0	30.0	30.0	30.0	34.0	35.0

All these results can be accepted since the legal Egyptian Standards (ES: 154-5/2005) state that the iodine values for the extracted fat should be in the range of 26.4–43.1 g 100 g^-1^. These results are not similar to those reported by another researcher who found that the iodine values of buffalo and cow ghee samples are 22.6 and 50.6 mg100 g^-1^, respectively (Pena-Serna and Restrepo-Betancur, 2020). It is considered that environmental conditions (such as temperature, origin, the composition of milk, processing condition, etc.) might have a significant effect on the composition of dairy products, and thereby the iodine value.

Typical analyses that are important include saponification number. This number indicates the average molecular weight. Results showed that the saponification number of all samples ranged from 220.0 to 237.0 mg KOH g^-1^. The saponification numbers of all fat samples (Table 3) are similar to the legal Egyptian Standards (ES: 154-5/2005), which ranges from 211.7 to 243.3 mg KOH g^-1^. Free fatty acids ranged from 24 to 33 (Table 3). These values agree with the legal Egyptian Standards (ES: 154-5/2005) for the fat, which ranges from 22.0 to 33.0. All obtained results of iodine value, saponification number, and free fatty acids are similar to the results of other studies (Celik and Bakirci, 2000; Fatouh et al., 2005; Özkanlı and Kaya, 2007; Zaidul et al., 2007; Samet-Bali et al., 2009; YR et al., 2012).

### 3.3 The protein fraction for dairy products

**Fig. 4:**
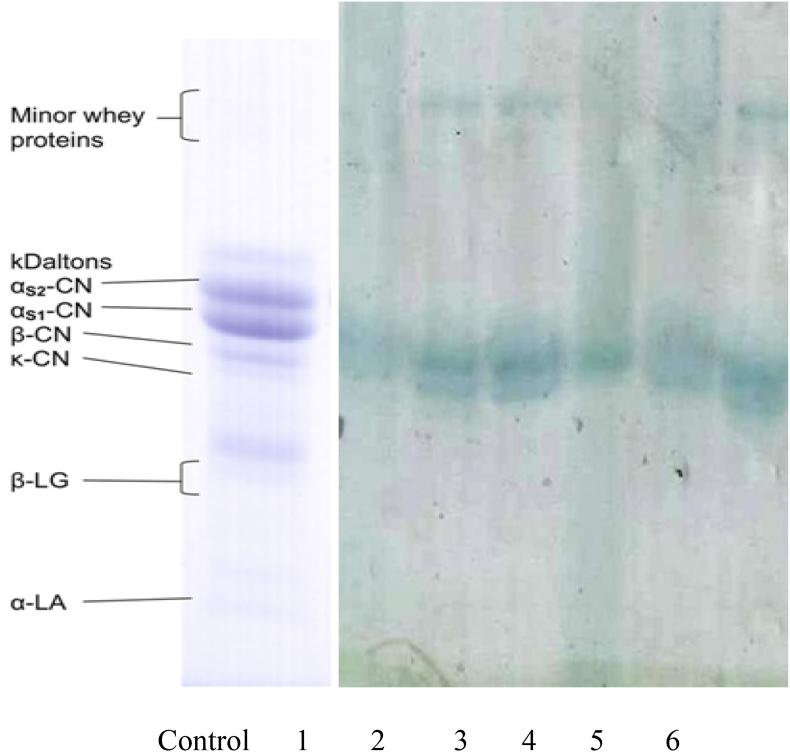
Polyacrylamide gel electrophoresis separation of the protein fractions determined in dairy products before and after shelf-life. Control: Reference profiles of bovine milk protein fractions (Paul and Van Hekken, 2011b) 1- Protein of sterilized milk before expiration. 2- Protein of sterilized milk after expiration. 3- Protein of yogurt before expiration. 4- Protein of yogurt after expiration. 5- Protein of cheese before expiration. 6- Protein of cheese after expiration.

The protein fractions were determined in sterilized milk, yogurt, and cheese before and after shelf-life using electrophoresis separation (Fig. 4). Results illustrate that no significant differences were found in casein fractions among different dairy products before and after expiration. Overall, the concentrations of intact casein were similar to control. Minor whey proteins were detected in all samples after expiration, which refers to small proteolysis. These results are in agreement with those reported in another study (Paul and Van Hekken, 2011a).

### 3.4 Fatty acids of cream and butter

The fatty acids profile in cream and butter before and after shelf-life is shown in Table 4. All fatty acids in cream samples did not have any significant differences but only minor differences were found. There was an increase in some fatty acids of cream after expiration but the largest increase was in C14:0, which elevated by approximately 0.4 mg g^-1^ after shelf-life of cream. On the other hand, there was a decrease in some fatty acids of cream after shelf-life, and the largest decrease was noticed in C18:0 (approximately 0.5 mg g^-1^ decrease).

**Table 4:** Gas chromatography fractionation of fat products (cream and butter) before and after shelf-life.

Fatty acids	Cream		Butter	
	Before	After	Before	After
C4:0	2.08	1.95	ND	0.18
C6:0	1.77	1.69	0.02	0.16
C8:0	1.10	1.10	0.05	0.53
C10:0	2.41	2.54	0.09	0.50
C11:0	0.27	0.26	N.D	N.D
C12:0	2.83	3.00	0.33	4.51
C14:0	9.34	9.76	1.39	2.78
C14:1	0.92	0.93	N.D	0.05
C15:0	0.96	0.98	0.10	0.17
C15:1	0.26	0.24	N.D	0.02
C16:0	31.28	31.08	39.37	38.13
C16:1	1.85	1.80	0.30	0.29
C17:0	0.52	0.56	0.14	0.18
C17:1	0.27	0.28	0.04	0.04
C18:0	9.75	9.29	4.81	6.05
C18:1	27.30	27.26	41.77	36.84
C18:2T	0.83	0.77	0.18	0.33
C18:2	3.15	3.42	10.50	8.25
C18:3n6	0.19	0.20	0.04	0.09
C18:3n3	0.49	0.40	0.23	0.19
C20:0	0.12	0.11	0.37	0.35
C20:1	0.23	0.22	0.16	0.12
C22:0	0.07	0.07	0.07	0.00

ND= Not detected.

In the butter sample, there was an increase after end of shelf-life in C6:0, C18:2T by 0.1 mg g^-1^, in C8:0 and C10:0 by 0.5 mg g^-1^, and in both C14:0, C18:0 about 1.4, 1.2 mg g^-1^, respectively. Also, a very noticeable increase in C12:0 (Lauric acid) about 4.2 mg g^-1^ was detected. There was also a shortage of both C16:0 and C18:2 by 1.2 mg g^-1^ and 2.3 mg g^-1^, respectively. A very noticeable lack of C18:1 (Oleic acid) was noticed at a rate of 5.0 mg g^-1^ (Table 4). Our results were similar to other studies.

### 3.5 Evaluation of the formulated creams

**Fig. 5:**
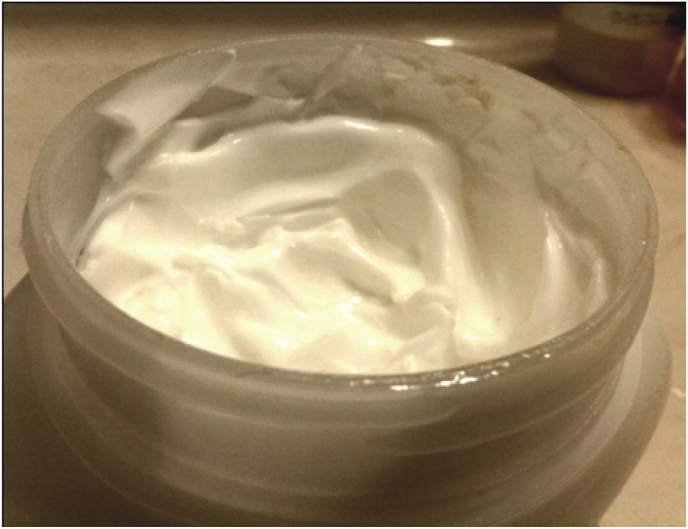
Formulated cream produced from expired dairy products fat (EDPF).

Formulated cream was evaluated for the parameters like visual appearance, spreadability, irritancy test, and pH. The appearance of cream was white color having a smooth greasy texture with no grainy particles (Fig. 5). Irritancy test was performed and there was no sign of redness and itching. All formulations were safe for topical application. The pH of the cream was found to be 5.63 ± 0.11, which is suitable for topical application on the skin. The prepared cream formula resulted in a good spreadability. The average spread circle diameter was 2.25 ± 0.19 cm.

## 4 Conclusion

According to the food legislation, all these products became not suitable for human consumption from the safety and quality point of view, but chemically they contain considerable amounts of undeteriorated or unrecompensed components, such as fat, protein, sugars, and minerals. No much differences were found in the composition of these products before and after shelf-life. Disposal of these products by burying them as wastes in the desert or the drainage channels represents a huge economic loss. As a result, it is useful to direct these substances into nonfood areas or nonfood industries. The study reveals that it is possible to formulate an effective cold cream containing expired dairy products and soap from expired dairy products fat.

## CRediT authorship contribution statement

**Nermeen N. Nasralla:** Conceptualization, Methodology, Software, Validation, Formal analysis, Investigation, Resources, Data curation, Writing – review & editing, Visualization, Supervision, Project administration, Funding acquisition. **Nanis H. Gomah:** Conceptualization, Methodology, Software, Validation, Formal analysis, Investigation, Resources, Data curation, Writing – review & editing, Visualization, Supervision, Project administration, Funding acquisition. **Morsy M. Aly:** Methodology, Software, Validation, Formal analysis, Investigation, Resources, Data curation. **Jelan A. Abdel-Aleem:** Methodology, Software, Validation, Formal analysis, Investigation, Resources, Data curation. **Ahmed R.A. Hammam:** Writing – original draft, Writing – review & editing. **Dina M. Osman:** Methodology, Software, Validation, Formal analysis, Investigation, Resources, Data curation. **Yaser M.A. El-Derwy:** Methodology, Software, Validation, Formal analysis, Investigation, Resources, Data curation.

## Declaration of competing interest

The authors declare that they have no known competing financial interests or personal relationships that could have appeared to influence the work reported in this paper.
